# Thermal Behavior of Polymeric and Other Advanced Materials

**DOI:** 10.3390/ma16072875

**Published:** 2023-04-04

**Authors:** Małgorzata Maciejewska, Magdalena Rogulska

**Affiliations:** Department of Polymer Chemistry, Institute of Chemical Sciences, Faculty of Chemistry, Maria Curie-Skłodowska University in Lublin, Gliniana 33, 20-614 Lublin, Poland; mrogulska@umcs.lublin.pl

Thermal Behavior of Polymeric and Other Advanced Materials is a recently open Special Issue in *Materials*, which aims to publish original articles, short communications, reviews, and mini-reviews covering the most recent progress in multiple thermal characterizations of diverse materials and provide a platform for scientists from various areas to present their research.

Thermal behavior can be regarded as a material’s response to being heated, cooled, or, sometimes, held isothermally. The determination of the relationship between temperature and specific physical properties of materials, especially thermal ones, is particularly important from the point of view of their usage and processing. Various thermal analysis techniques, such as thermogravimetry (TG), differential thermal analysis (DTA), and differential scanning calorimetry (DSC), can be used to achieve this goal. As is well known, advanced materials are often expected to withstand extremely high or extremely low temperatures. Application of the DTA and DSC allows one to determine temperatures and enthalpies of phase transformations as well as heat capacities in a temperature range from −150 to 1500 °C. In turn, the TG provides information on the thermal stability of materials and their decomposition process. Thermal techniques are also of great importance because they enable the evaluation of chemical purity of many compounds, precursors, and ultimate products. Furthermore, detailed investigation of the thermal behavior of divergent materials creates a possibility to improve their properties and achieve more effective ones. A deep insight into thermal properties as well as the recognition of changes in these properties during exploitation results in the expansion of the area of their application.

Additionally, thermal properties are of paramount importance in the environmental aspects of the investigation, including the combustion and recycling of polymeric materials, the thermal use of polymer waste with energy recovery, photovoltaic and thermal insulation materials, and many other significant issues.

The research interest in the Thermal Behavior of Polymeric and Other Advanced Materials includes, but is not limited to, the following: thermal stability of polymers and other advanced materials, thermogravimetry (TG) and evolved gas analysis, differential scanning calorimetry (DSC), differential thermal analysis (DTA), dynamic mechanical thermal analysis (DMTA), decomposition of polymers and composites, thermal energy storage, thermal insulation materials, and thermal recycling of polymeric materials.

